# Research of Flexible Assembly Job-Shop Batch–Scheduling Problem Based on Improved Artificial Bee Colony

**DOI:** 10.3389/fbioe.2022.909548

**Published:** 2022-08-16

**Authors:** Xiulin Li, Jiansha Lu, Chenxi Yang, Jiale Wang

**Affiliations:** ^1^ Department of Logistics Management and Engineering, Zhejiang Gongshang University, Hangzhou, China; ^2^ Institute of Industrial Engineering, Zhejiang University of Technology, Hangzhou, China

**Keywords:** flexible assembly job shop, batch splitting, batch scheduling, lot streaming, consistent size, unequal size, artificial bee colony

## Abstract

This study examined the flexible assembly job-shop scheduling problem with lot streaming (FAJSP-LS), common in multivariety and small-batch production, such as household electrical appliances. In FAJSP-LS, an assembly stage is appended to the flexible job shop, and jobs in the first stage are processed in a large batch to reduce switching costs, while leading to more waiting time, especially during the assembly stage. This article considered splitting the batch into a few sub-batches of unequal and consistent sizes to allow jobs to efficiently pass the two-stage system. With this objective, the problem was modeled as a mixed-integer linear program comprising the following two subproblems: batch splitting and batch scheduling. As the integrated problem is NP-hard, the improved bioinspired algorithm based on an artificial bee colony was proposed, including a four-layer chromosome–encoding structure to describe the solution, as well as an optimization strategy utilizing different bee colonies to synchronously solve this two-stage problem. To examine the algorithm’s efficiency, a benchmark case was used to show that better solutions can be acquired with the improved algorithm regardless of whether the batch was split into equal or unequal sizes. To promote practical implementation, the algorithm was applied to a real case refrigerator workshop and showed better performance on time efficiency when jobs were split into unequal sizes compared to jobs without splitting or splitting into equal sizes.

## 1 Introduction

An assembly job shop is a two-stage production structure in that an assembly stage is appended to a job shop. Once the assembly stage is appended, the job-shop scheduling problem becomes the assembly job-shop problem (AJSP) (Wong and Ngan, 2013). In AJSP, some jobs are dependent so that they can be completed directly after the machining stage. In contrast, some jobs are independent and need to enter the assembly buffer waiting for specific jobs with assembly relationships according to the bill of material. A flexible job shop is a generalized version of the job shop and is more common. The AJSP will expand to a flexible assembly jobs-hop scheduling problem (FAJSP) when the assembly stage is appended to a flexible job shop.

**FIGURE 1:**
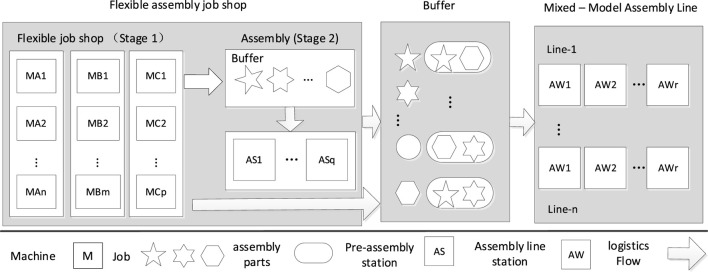
Structure of flexible assembly job-shop with following mixed-model assembly line.

This study was inspired by a real-world case in a refrigerator factory, which has the flexible assembly job-shop structure shown in Figure 1. This structure usually exists in the processing and preassembly stage before multivariety and small-batch mixed-model assembly line, such as the household electrical appliance production. As the TAKT time upstream is significantly shorter than the downstream mixed-model line, the job is usually processed in a batch to reduce the total setup time. Large batches are accessible to the backlog of work in process (WIP) among machines if the batch is wholly moved, especially for the independent jobs when waiting for assembly parts. Lot streaming (LS) is a technique that allows splitting a large batch into a few smaller sub-batches and produce on parallel machines to smooth the following demand of multivariety jobs from the assembly line and shorten the flow time of each job to reduce WIP. When LS is applied, the FAJSP expands to FAJSP with LS (FAJSP-LS), which includes the following four decisions: 1) the quantity of sub-batch to each job, 2) the size of each sub-batch, 3) the machine selection for each operation of sub-batch, and 4) sequencing of operations on each machine. Combining with assembly and lot-splitting, the FAJSP-LS is more complicated than FJSP, which was proved to be NP-hard.

As the fundamental problem of FAJSP-LS, AJSP was first studied in the 1960s to solve a multilevel assembly scheduling problem under a random environment (Pereira et al., 2011). Due to the complexity of kit constraints in assembly, heuristic rules of distribution and scheduling are primarily used to solve them in an early stage of research. For example, the production structure of an assembly workshop, including single-part and multipart products, was studied, and a hybrid rule based on the shortest processing time (SPT) and assembly jobs first with SPT as a tie-breaker (Asmf-spt) was proposed to optimize the objectives of tardiness and process time (Huang et al., 1984). Thiagarajan et al. (2005) proposed a series of heuristic rules for distribution scheduling to optimize the comprehensive objectives such as weighted tardiness and process time. Omkumar et al. (2009) proposed a heuristic method based on an ant colony algorithm to solve the multilevel assembly-job-scheduling problem and compared it with various rules to verify the performance advantages of the algorithm. Compared with the existing production system, the classical AJSP problem was simplified in two aspects. First, the flexibility of machine selection and process flow was not considered in the processing stage; second, the impact of a large batch on the operation time and inventory in actual production was not considered.

To solve the flexibility problem, the FAJSP is proposed as an AJSP extension. Benjaafar and Ramakrishnan. (1996) and Zhang et al. (2020) defined and summarized the flexibility in production as operation, process, and sequence flexibility. Among these, process flexibility is the most common; that is, the job process can be processed on multiple optional processing machines. However, before deciding the processing sequence of each process on the machine, it is necessary to allocate the process to a specific machine. Therefore, FAJSP is also regarded as an integration of AJSP and integrated planning and scheduling problem (Nourali et al., 2012; Zhang et al., 2020). Nourali et al. (2012) defined the FAJSP and established a mixed-integer programming model considering makespan, and designed a PSO to solve it. Zhang and Wang (2018) and Zhang et al. (2020) studied FAJSP with component sharing, established a constraint programming model and mixed-integer programming model, and designed scheduling rules and distributed an ant colony algorithm. Wu et al. (2019) considered the FAJSP based on the distributed workshop architecture and proposed a model considering tardiness, production, and transportation costs and designed a genetic algorithm to solve it. Lin et al. (2022) studied FAJSP with a tight job based on a genetic algorithm.

To solve the batch processing problem, LS was used to split the product batch into production/transfer sub-batches and smoothly transition to a multistage production process through sorting and coordination sub-batches to shorten the production cycle. In terms of sub-batch size, the batch-splitting strategy can be divided into that of equal and variable size (Trietsch and Baker, 1993) and into consistently sized (the sub-batch size of the different job is different but remains unchanged during processing) and variably sized (the sub-batch size of the different job is different but can be changed when moving to next machine) (Martin, 2009). Low et al. (2004) proved that the equal-sized division method performs better in optimizing the time-related target. Similar studies also show that batch splitting can effectively reduce the system makespan and reduce WIP (Kalir and Sarin, 2000). However, Low (2004) also pointed out that too many batches will lead to a decline in time performance. That means the connection between the batches’ quantity and completion time is U-shaped. Both too large and too small quantity will lead to the reduction in time performance. This phenomenon is more pronounced when there are assembly and flexible constraints in the production process. Chan et al. (2008) studied LS with AJSP (AJSP-LS) for the first time and solved equal and unequal problems using a genetic algorithm. Furthermore, Wong and Ngan (2013) continued this study and designed a hybrid genetic algorithm and hybrid particle swarm. Ba et al. (2015) considered taking the equal-size strategy to divide all jobs and use PSO to optimize the scheduling of divided sub-batches.

In summary, among the reviewed literature, most studies focus on the flexibility problem or batch processing separately. However, an integrated problem, flexible assembly job shop with LS (FAJSP-LS) that includes these two problems is closer to the reality in processing-assembly production. Thus, Zeng et al. (2019) studied the FAJSP-LS and designed a hierarchical iteration algorithm that first uses improved GA to solve the batch-splitting subproblem and then distribution rules such as first come first served (FCFS) and operation due date to solve the batch-scheduling subproblem. However, compared with the upper-layer batch–splitting problem, the lower layer batch-scheduling problem, which includes machine assignment and sub-batch sequencing, has a larger search space. Solutions can be acquired quickly with distribution rules, but at the expense of performance. Other hierarchical iteration or integrated algorithms in the reviewed literature had shown good performance on AJSP-LS and found that equal-size LS strategy is better than unequal one ([Chan et al., 2008; Wong and Ngan. (2013)]. However, the conclusion may change under the FAJSP environment, because flexible machine choices are allowed for each operation of the sub-batch. In addition, nearly all studies restricted the assembly to be processed only after all the sub-batches of assembly jobs are ready, which potentially increased the makespan, and WIP then affects the conclusion.

To the best of our knowledge, the FAJSP-LS has been rarely studied. Hence, in this article, the problem was described in detail, and a mathematical model was proposed based on unequal and consistent size that sub-batches of the same job may have unequal size and the size had to be maintained during the entire processing route (Novas, 2019). Considering unequal size, this study also relaxed the restriction that sub-batches can be assembled even if they are not the same size. For example, jobs A and B have assembly connections, and the size of sub-batch *A*_*i*_ of job A and *B*_*j*_ of job B are 100 and 200, respectively. One hundred pieces of both A and B can be assembled directly once these two sub-batches pass through the first stage, and the other 100 pieces of B will be left waiting for more A to assemble. Applying this rule can increase flexibility to batch splitting and significantly reduce flow time and WIP, but at the cost of more complexity to batch scheduling.

Hence, in this study, a global optimization algorithm was designed to solve the integrated problem directly based on the multipopulation collaboration mechanism of the artificial bee colony algorithm (ABC). ABC is a bioinspired algorithm that is easily tailored to a new problem and obtains near-optimal solutions (O Kheirandish et al., 2015; Xie et al., 2022). ABC was inspired by the foraging behavior of bee colony and had shown better performance than other bioinspired algorithms such as particle swarm optimization and genetic algorithm (Karaboga and Akay, 2009). Since it was proposed in 2005, ABC has been widely used to tackle combination optimization problems, such as flexible job-shop scheduling problems and flow shop optimization (Gong et al., 2020; Meng et al., 2018; Li et al., 2017). Most of the existing algorithms take distributed strategies for solving these two subproblems separately or iteratively (Chan et al., 2008), or take integration strategies but simplify one subproblem by using heuristic rules or batch division rules (Ba et al., 2015; Zeng et al., 2019). In study article, a four-layer chromosome–encoding method was designed for this algorithm to describe the two-stage problem, and an optimization strategy was designed to assign these two subproblems to different populations and then optimized as a whole to acquire ideal splitting and effective scheduling synchronously. Computational experiments were performed to examine the integrated optimization performance for this kind of two-stage problems and test the performance of splitting batches into equal and unequal sizes. Furthermore, a real refrigerator production case tests the effectiveness of unequal-size batch splitting with a minimum average flow time.

**Table:** 

**Parameters**	**Description**
*m*	Total number of machines
*n*	Total number of jobs
*n*_*i*_	Total number of options of job *Ji*
$Am_{i}$	Total amount of job *Ji*
*Lp*	Minimum size of sub-batch
**Variables**	**Description**
$L_{i}$	Total number of sub-batches of job *Ji, (i=1,…, n)*
$Am_{ip}$	Volume of the *p*th sub-batch of job *Ji*
$E_{ipjk}$	The completion time of operation *j* of *p*th sub-batch of job *Ji* on machine $M_{k}$ *(i=1,…, n; j=1,…, n*_*i*_ *; p=1,…, L*_*i*_ *; k=1,…, m)*
$E_{ip}$	The completion time of the *p*th sub-batch of job *Ji*
$F_{ip}$	The flow time of the *p*th sub-batch of job *Ji*
$F_{ipjk}$	The flowtime of the *j*th operation of *p*th sub-batch of job *Ji* on machine $M_{k}$
$PT_{ijk}$	The preparation time for the *j*th operation of job *Ji* on the machine $M_{k}$
$W_{ipjk}$	The waiting time of the *j*th operation of *p*th sub-batch of job *Ji* on machine $M_{k}$
${\text{A}}_{\text{ipjk}}$	The time when the *j*th operation of *p*th sub-batch of job *Ji* arrive machine $M_{k}$
$B_{ipjk}$	The time when the *j*th operation of *p*th sub-batch of job *Ji* start processing on machine $M_{k}$
$P_{ijk}$	The processing time of *j*th operation of job *Ji* on machine $M_{k}$
$\alpha_{ii\prime }^{k}$	$\{\begin{array}{c} 1\\ 0 \end{array}\begin{array}{c} \;\;\text{Job }J_{i}\text{ and }J_{i\prime }\text{ processed continuously on }M_{k}\text{ are of the same type}\\ \text{else }\;\;\;\;\;\;\;\;\;\;\;\;\;\;\;\;\;\;\;\;\;\;\;\;\;\; \end{array}$
$\gamma_{i}^{i\prime }$	$\{\begin{array}{c} 1\;\\ 0 \end{array}\begin{array}{c} \text{job}\;Ji\text{ and job}\;Ji\prime \text{ have assembling relation}\\ \text{else} \end{array}$
$X_{ipjk}$	$\{\begin{array}{c} 1\\ 0 \end{array}\begin{array}{c} \;\;\text{the }jth\;\text{operation of }pth\;\text{sub}-\text{batch of job }Ji\text{ is processed on }M_{k}\\ \text{else} \end{array}$

## 2 Problem Description and Formulation

### 2.1 Problem Description

The FAJSP-LS can be described as follows. The job shop has *m* machines that can process *n* kinds of jobs. Each job contains *n*_*i*_ processes that can be processed on at least one machine with different processing times. There is an assembling relation between at least two or more jobs. Each number of jobs can be split into multiple sub-batches with different sizes and keeping the size during processing and transport. In actual production, assembly jobs are generally transferred in one circulation box or according to the number of KanBan. Therefore, the minimum number of transport units is defined as *Lp*, and the size of the sub-batch is an integral multiple of *Lp*. The objective is to minimize each sub-batch’s average flow time, reflecting the sub-batch transfer efficiency and WIP level (Huang et al., 2010). The flow time of a job on a machine mainly includes the waiting, setup, and processing times, and the difference between the completion time and arrival time. When two sub-batches continuously processed on the same machine belong to the same job, the preparation time is not required. Other main assumptions are as follows:

1) The volume of jobs with assembling relation is equal.
2) All sub-batches can be processed at the moment zero.
3) Any machine can only process one sub-batch at a time, and processing cannot be interrupted.
4) There is no transferring time and cost between machines.
5) There is no assembly time during the assembly stage.
6) The buffer between machines or stages is infinite.

### 2.2 Problem Formulation

Notations:

$$FT=\min\left(\left(F_{1}+F_{2}\right)/\sum_{i=1}^{n}L_{i}\right)$$ (1)

$$F_{1}=\sum_{i=1}^{n}\sum_{p=1}^{L_{i}}\sum_{j=1}^{n_{i}}\sum_{k=1}^{m}F_{ipjk}$$ (2)

$$F_{2}=\sum_{i=1}^{n}\sum_{i\prime =1}^{n}\left|\left(\overset{L_{i}}{\underset{p=1}{\sum }}E_{ipn_{i}k}-\overset{L_{i\prime }}{\underset{p\prime =1}{\sum }}E_{i\prime p\prime n_{i\prime }k\prime }\right)\right|\times \gamma_{i}^{i\prime }/2,\;\;k=1,2,...m$$ (3)

$$\begin{array}{l} F_{ipjk}=W_{ipjk}+P_{ipjk}+PT_{ijk}\left(1-\alpha_{ii^{\prime }}^{k}\right)\\ F_{ip1k}=P_{i1k} \end{array}$$ (4)

$$\begin{array}{l} i,i\prime =1,2,...,n;j=2,...,n_{i};p=1,2,...,L_{i};k_{1},k_{2}=1,2,...,m\\ E_{ipjk}=A_{ipjk}+F_{ipjk} \end{array}$$ (5)

$$\begin{array}{l} i=1,2,...,n;\;\;p=1,2,...,L_{i};\;j=1,2,...,n_{i};\;k=1,2,...,m\\ W_{ipjk}=B_{ipjk}-A_{ipjk}\\ A_{ipjk}=E_{ip\left(j-1\right)k\prime },\;\;B_{ip1k}=A_{ip1k}; \end{array}$$ (6)

$$\begin{array}{l} i=1,2,...,n;j=1,2,...,n_{i};p=1,2,...,L_{i};k,k\prime =1,2,...,m\\ P_{ipjk}=WT_{ij}^{k}\times Am_{p}^{i} \end{array}$$ (7)

$$\begin{array}{l} i=1,2,...,n;j=1,2,...,n_{i};k=1,2,...,m;p=1,2,...,L_{i}\\ Am_{i}=\overset{L_{i}}{\underset{p=1}{\sum }}Am_{ip},Am_{ip}=\gamma \times Lp,\\ \gamma \in N,1\leq \gamma \leq \left(Am_{i}\right)/Lp \end{array}$$ (8)

Eq. 1 is the objective of average flow time minimization. $F_{1}$ is the total flow time of all sub-batches on the job-shop stage, and $F_{2}$ the total flow time of all sub-batches during the assembly stage. Eq. 2 is the calculation of $F_{1}$ ; Eq. 3 is the calculation of $F_{2}$ ; Eq. 4 indicates that the flow time of sub-batch on machine is equal to the sum of the waiting, setup, and processing times; Eq. 5 indicates the calculation of completion time of sub-batch; Eq. 6 defines the waiting time of sub-batch; Eq. 7 defines the processing time of sub-batch; and Eq. 8 guarantee that the sum of all sub-batch is an integral multiple of the *Lp.*

## 3 Proposed Improved Artificial Bee Colony Algorithm

### 3.1 Framework of the Algorithm

**FIGURE 2:**
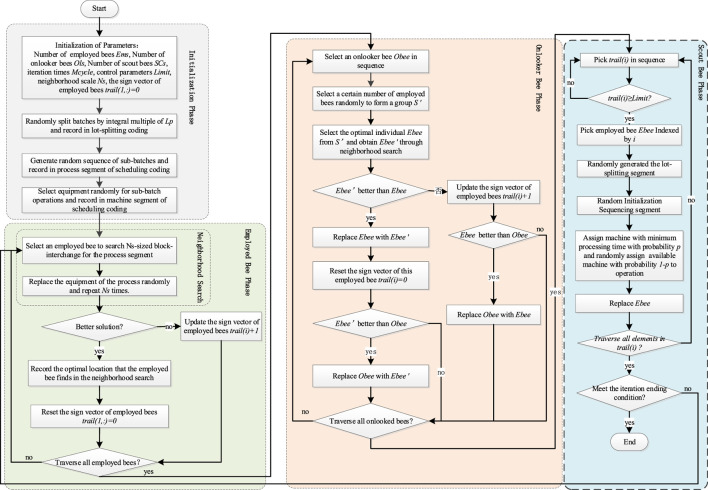
Framework of improved algorithm based on an artificial bee colony.

As a bioinspired algorithm, ABC has been studied and applied widely, and it shows a better performance in the combination optimization problem (Karaboga et al., 2014; Meng et al., 2018; Gong et al., 2020). Based on the multipopulation collaboration mechanism of the ABC algorithm, the batch-scheduling subproblem, which has a more extensive solution space, was assigned to employ bee and onlooker bee to optimize, while the batch-splitting subproblem was optimized by the scout bee. The critical control parameter *limit* was designed for employer bee and onlooker bee and storage in vector *trail* to enlarge the global optimization capability. A neighborhood search is designed for the employed bee to strengthen the local optimization of the employed bee. Moreover, a similar design is adopted by the onlooker bee to choose an employed bee from a random temp group *S’* instead of an employed bee. Figure 2 shows the algorithm architecture.

### 3.2 Detailed Design of the Algorithm

This part includes a detailed encoding, initialization, and scout bee phase design. The employed bee and onlooker bee design can be found in former research (Li et al., 2017).

#### 3.2.1 Chromosome Encoding

FAJSP-LS is typical discrete optimization problem, and it is necessary to put up an encoding method to structure its solution. It includes two subproblems with four decisions: quantity of sub-batches for each job and lot size for each sub-batch in the batch-splitting subproblem, machine arrangement and processing sequence on specific machine in sub-batch-scheduling subproblem. To encode the solution completely, a four-layer chromosome–encoding structure was proposed to describe the solution, and each segment was encoded using positive integers that record the real value of decision variable (xie, 2022). These segments are recorded as a one-dimensional array of a different size. The LA and LB segments in the first two layers are for the batch-splitting subproblem. LA is the sub-batch quantity segment with *n* elements, *n* is the number of jobs, and the *i*th element $\left\{L_{i},i=1,2,...,n\right\}$ brepresents the sub-batch quantity of job *J*_*i*_. The LB segment represents the size of each sub-batch, which can be indexed according to the cumulative number of sub-batches. Assume *SL*_*q*_ is the *q*th sub-batch in all sub-batches, $q=1,2,...,\overset{n}{\underset{i=1}{\sum }}L_{i}$ . The *q*th element *Am*_*ip*_ in LB represents the quantity of *SL*_*q*_, which is also the *p*th sub-batch of job *J*_*i*_, The correlation of *q* and *p* is described as Eq. 9:

$$q=\overset{i-1}{\underset{i=1}{\sum }}L_{i,}+p,\;1\leq p\leq L_{i}$$ (9)

The JA and JB segments in the last two layers are for sub-batch scheduling. JA represents the operations’ sequence of each sub-batch, and JB represents the machine assignment for each operation. When each sub-batch is regarded as a job, then the regular encoding method for FJSP (Li et al., 2011) could be adopted in these two segments. Assume the size of JA *is L*_*JA*_ as shown in Eq. 10:

$$L_{JA}=\overset{n}{\underset{i=1}{\sum }}\overset{L_{i}}{\underset{p=1}{\sum }}n_{i}*p$$ (10)

**FIGURE 3:**
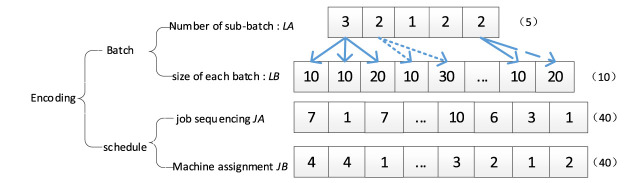
Encoding example.

Figure 3 shows an example of problem coding. The number of machines *m = 4*, the type of jobs *n* = 5, and each job has four *n*_*i*_ operation*s.* The LA segment represents the number of sub-batches of each job. The LB segment represents the size of each sub-batch, which can be indexed according to the job number. For example, job *J*_*1*_ has 40 pieces to be processed, the number of sub-batches is three, and the sizes are 10, 10, and 20 pieces, respectively. The encoding of job sequencing JA shows the operations sequence of each sub-batch. In this segment, the first element seven is the first operation of the 1st sub-batch of *J*_*4*_, and the second element one is the first operation of the 1st sub-batch of *J*_*1.*_ They are all assigned to machine four according to the machine assignment segment JB, and the operation of *J*_*4*_ is processed ahead of the operation of *J*_*1.*_ The second seven in JA is the second operation of the 1st sub-batch of *J*_*4*_.

The advantages of the proposed four-layer chromosome encoding are as follows: 1) high flexibility to meet the uncertain size of LB, JA, and JB segments. The size of LA is known as the number of jobs, once the LA segment is determined, the size of the other three segments can be acquired. 2) The structure can ensure the whole search space, and any solution can be encoded into only one chromosome; correspondingly, any one chromosome can also be decoded into only one legal solution. 3) The four-segment structure shows the relevance between segments in a more intuitive way.

#### 3.2.2 Population Initialization of Algorithm

The initialization phase includes batch segment and scheduling segment initialization as follows:

1) Generate the batch segment code; the size of every sub-batch is the integral multiple times of *Lp.* Then the maximum number of sub-batches is $Am_{i}/Lp$ for the job *i*. Then, a random *L*_*i*_ is generated with the constraint, $L_{i}=1,2,...,Am_{i}/Lp]$ and it generates the coding segment LA*.*
2) In random split the job batch into *L*_*i*_ sub-batches, make sure the size of each sub-batch is an integral multiple of *Lp*, and generate the coding segment LB.
3) Regard the sub-batch as a new job and randomly generate the scheduling sequence of operations of these new jobs and encoding segment JA.
4) In random assign operations to available machines and generate encoding segment JB.
5) Repeat the above steps $\overset{n}{\underset{i=1}{\sum }}Am_{i}/Lp$ times to finish the initialization of the employed and onlooker bees.

#### 3.2.3 Scout Bee Algorithm

The scout bee algorithm undertakes the optimization for batch splitting. The employed and onlooker bees whose *trail* fit the control parameter *limit* will trigger the scout bee algorithm and generate a new batch-splitting solution. The steps, which are similar to initialization, are as follows:

1) Follow the first two steps of population initialization that randomly generate the number of sub-batches of each job.
2) Follow the third step of population initialization to structure the job sequencing segment, assign a machine with minimum processing time with probability *p*, and randomly assign an available machine with probability *1-p* to the corresponding operation per batch.

## 4 Experiments and Results

In this section, the performance of the proposed algorithm is separately tested on FJSP, FAJSP, and FAJSP-LS cases. Furthermore, a design of the experimental method is used to optimize the parameters.

### 4.1 Parameter Setting

The proposed ABC algorithm has the following three main parameters: population size (*Ps*), the maximum number of trials *limit*, and probability *p* that assigns the machine with minimum processing time to an operation in scout bee stage. Among them, *Ps* and *limit* are intrinsic parameters of ABC, and limit is the key parameter that decides the global performance of the algorithm and impacts the optimization capability of the batch-splitting subproblem. *P* is a secondary parameter adopted to improve local optimization capability on batch-scheduling subproblem.

Population size *Ps* is usually set as a specific number (Gong et al., 2020; Li et al., 2020). However, it should be associated with the scale of problem to be solved. The maximum number of sub-batches *TL* can be described as *TL* = $\overset{n}{\underset{i=1}{\sum }}Am_{i}/Lp$ and the *Ps* is designed to four levels*; limit* is set as (4, 6, 8, 10) according to the existing research (Li et al., 2011; Xie et al., 2020). The parameters are shown in Table 1.

**TABLE 1:** Parameters table.

Parameter	Level			
	1	2	3	4
*Ps*	TL	1.5 × TL	2 × TL	2.5 × TL
*limit*	4	6	8	10
*p*	0.2	0.4	0.6	0.8

**FIGURE 4:**
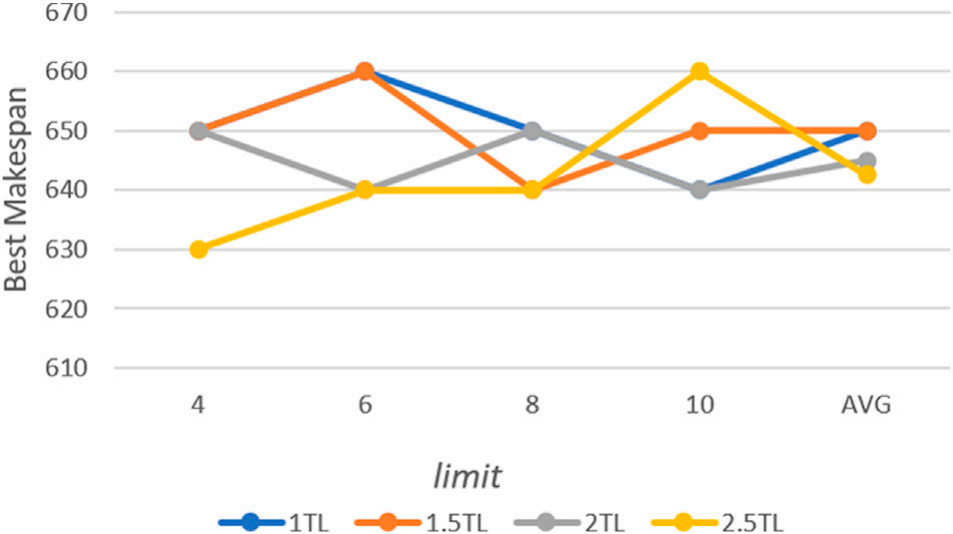
Trend of best solution with *Ps* and *limit*.

**FIGURE 5:**
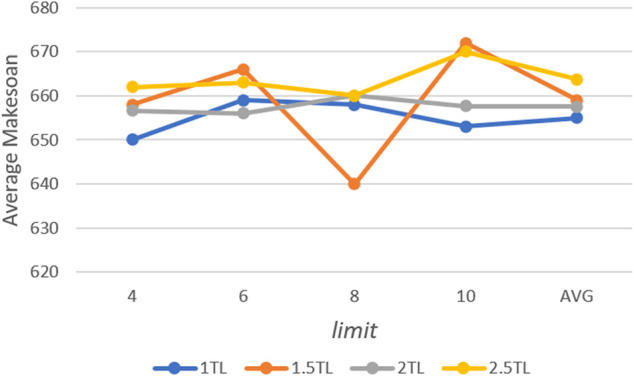
Trend of average solution with *Ps* and *limit*.

The middle scale 10 × 10 case from Kacem benchmark (Kacem et al., 2002) is used to test the parameters. The max iteration times are 500. First, the *Ps* and *limit* are tested. For each possible configuration, the proposed algorithm is run 10 times independently. The best and average solutions’ trends are show in Figures 4 and 5. Avg is the average value of different *limit* for a specific *Ps.*

**FIGURE 6:**
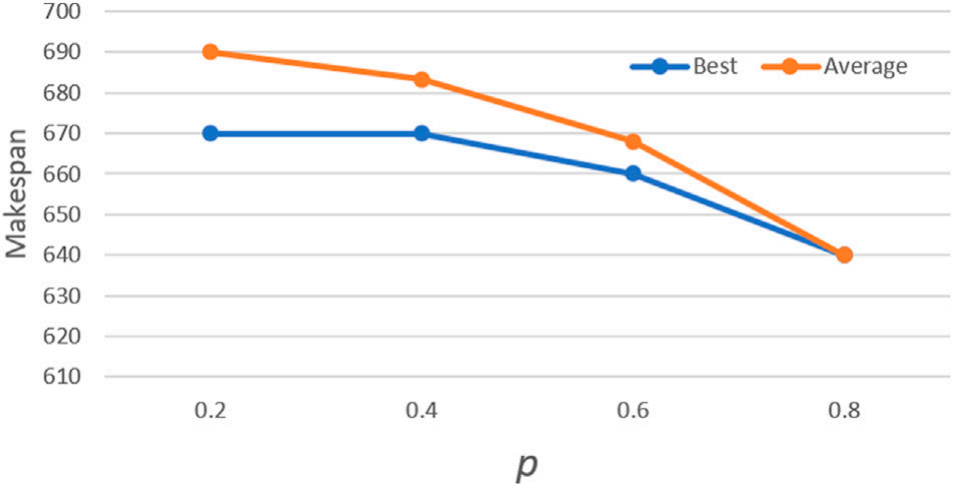
Trend of *P.*

According to the results, the population size has an important impact on performance. Better solutions could be acquired with the increase in population size, however, more computation time is needed. The similar result can be acquired when *Ps* is 1.5 × TL, and it is also the most stable choice for the solution. Moreover, a trend can be observed that the lower *limit* shows better performance when *Ps* is larger, and vice versa. That means it needs more chances to avoid falling into local optimal when it has a larger population. For *limit*, eight is the most appropriate value. The algorithm is run 10 times for each value of *P with Ps =* 1.5 × TL and *limit* = 8. Furthermore, *P* is set as 0.8 according to the result in Figure 6.

### 4.2 Algorithm Performance Test

**FIGURE 7:**
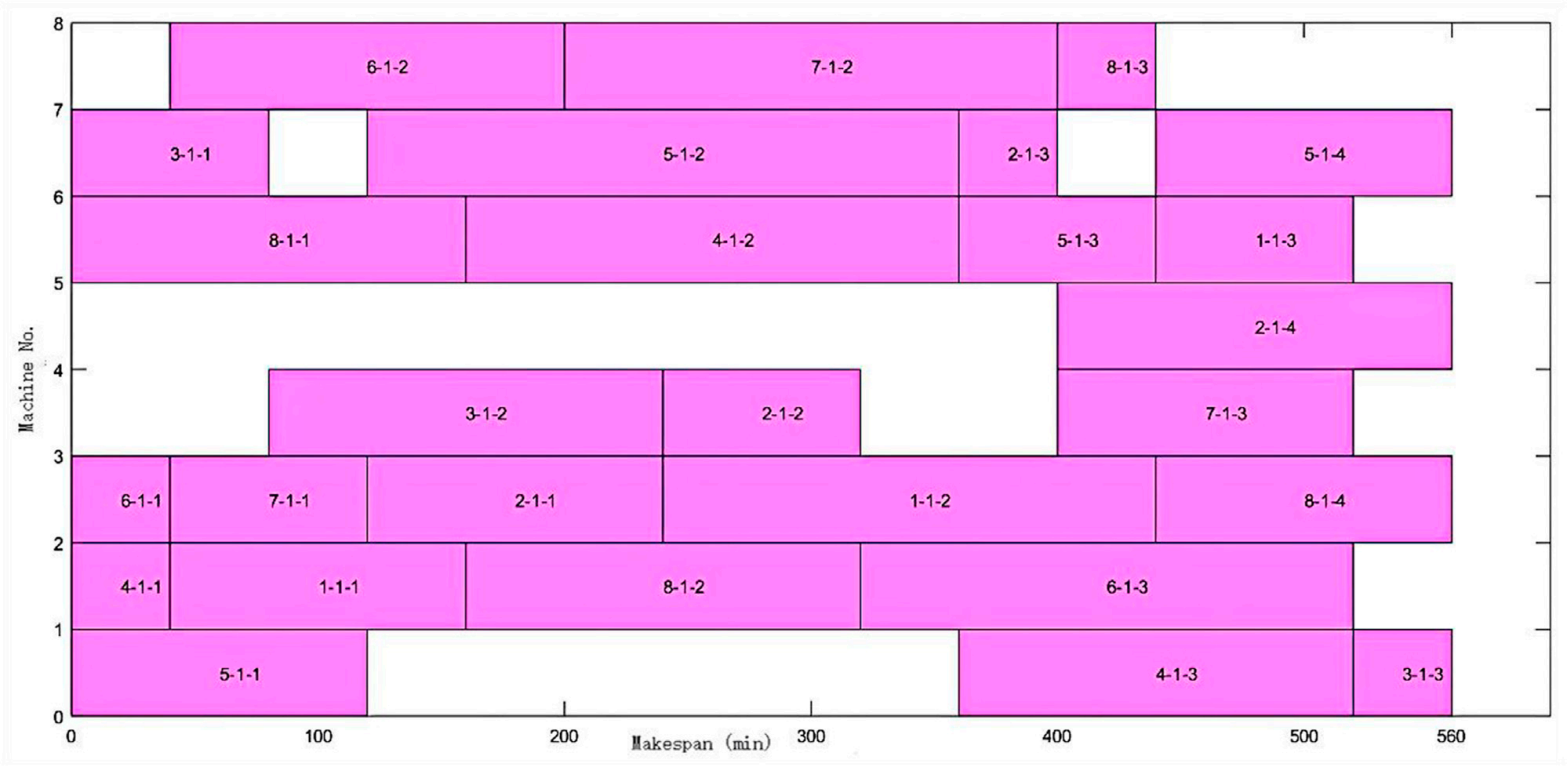
Kacem 8 × 8 Gantt chart without batch splitting.

FAJSP-LS with the unequal and consistent batch size is a new problem, and there is no benchmark for testifying available. As the fundamental problem of FAJSP-LS, the FJSP benchmark can be used to verify the integrated performance for splitting and scheduling of the proposed algorithm. The 8 × 8 case from Kacem benchmark (Kacem et al., 2002) was used. Without batch splitting, the near-optimal result obtained by the proposed algorithm in this article is 560, the same as the best results obtained in former research (Yazdani et al., 2010; Lu et al., 2012). Figure 7 shows the scheduling Gantt Chart.

Besides, 4 × 6 FJSP from Kacem is used to verify the algorithm’s equal and unequal splitting performance, respectively. The results are shown in Table 2.

**TABLE 2:** 4 × 6 Comparison of batch splitting and scheduling results.

	No. of sub-batch (equal size)	Makespan (equal size)	No. of sub-batch (unequal size)	Makespan (unequal size)
Sun et al. (2008)	13	87	/	/
Bai et al. (2010)	8	90	8	84
Xu et al. (2016)	10	86	9	83
This article	8	80	10	78

**FIGURE 8:**
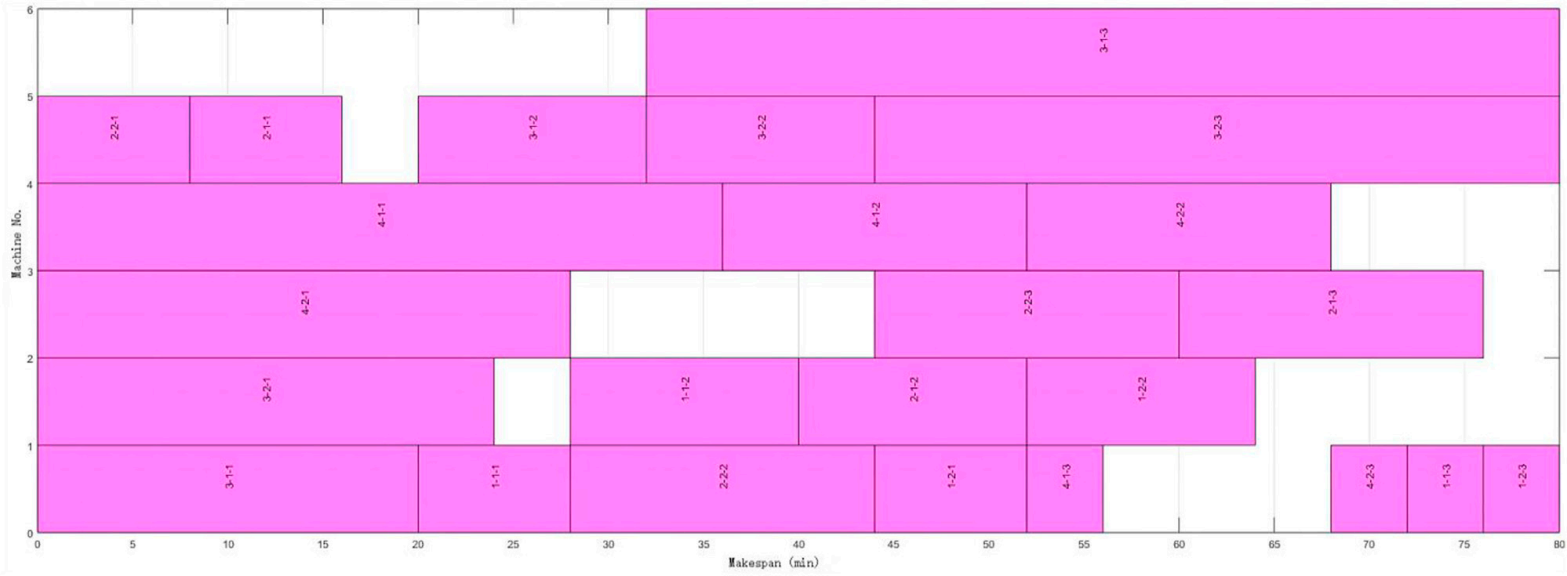
4 × 6 Gantt chart of equal-size batch splitting and scheduling.

**FIGURE 9:**
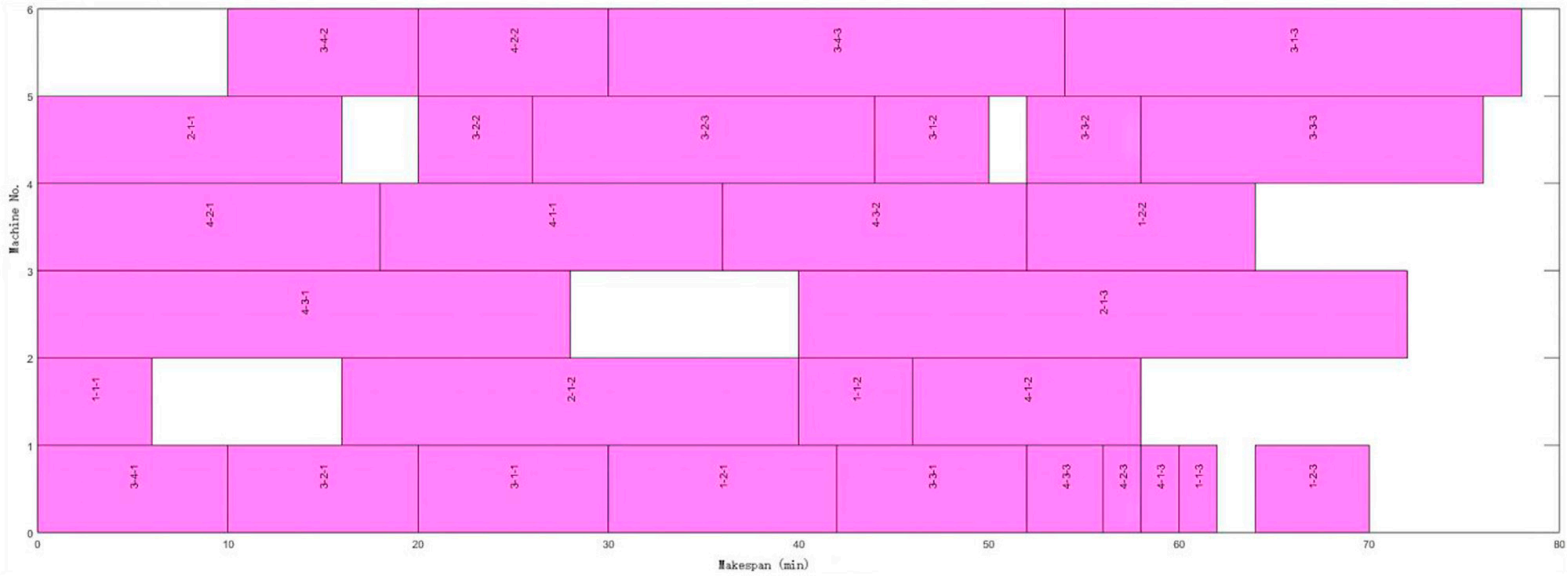
4 × 6 Gantt chart of unequal-size batch splitting and scheduling.

The proposed algorithm performs well in both equal and unequal-size splitting compared with the above studies. Better performance can be obtained with a smaller number of sub-batches. Figures 8 and 9 are Gantt charts of equal size and unequal-size scenarios, respectively.

### 4.3 Case Analysis

The proposed algorithm is applied to a confirmed case of a refrigerator shell parts production workshop, a typical flexible job-shop combined with an assembly stage. The flexible job-shop produced the upper bar A and B, the lower bar A and B, the U-shell A and B, the backplate A and B, the front shell A, the left plate A, and the right plate A. The job set is denoted as $J=\left\{J_{i}\right\},i=1,2...11$ . Machine set is denoted as $M=\left\{M_{j}\right\},j=1,2...10$ . The jobs with assembly constraints are $\left(J_{1},J_{2}\right)$ , $\left(J_{3},J_{4}\right)$ , and $\left(J_{10},J_{11}\right)$ . The minimum size of the sub-batch is Lp=100 , and the preparation time is 100. Table 3 shows the production plan. Table 4 is the processing time matrix of operations on each machine.

**TABLE 3:** Production plan.

Model	J1	J2	J3	J4	J5	J6	J7	J8	J9	J10	J11
Quantity	400	400	300	500	400	200	200	400	400	600	500

**TABLE 4:** Processing time matrix.

Job	Operation	Processing machine									
		*M*_*1*_	*M*_*2*_	*M*_*3*_	*M*_*4*_	*M*_*5*_	*M*_*6*_	*M*_*7*_	*M*_*8*_	*M*_*9*_	*M*_*10*_
*J*_*1*_	*O*_*11*_	6.5	7.2	6.5	8	—	—	—	—	—	—
	*O*_*12*_	—	—	—	—	7	6	6	6.5	—	—
	*O*_*13*_	—	—	—	—	7.5	7	8	6	—	—
*J*_*2*_	*O*_*21*_	7	7	7	6	—	—	—	—	—	—
	*O*_*22*_	—	—	—	—	8	8.4	7.6	8	—	—
	*O*_*23*_	—	—	—	—	7	5	6	7	—	—
*J*_*3*_	*O*_*31*_	5.5	5.5	7	6	—	—	—	—	—	—
	*O*_*32*_	—	—	—	—	4	4	5	4	—	—
	*O*_*33*_	—	—	—	—	5	5.5	6	6	—	—
*J*_*4*_	*O*_*41*_	8	7.7	8	7.5	—	—	—	—	—	—
	*O*_*42*_	—	—	—	—	5	5	7	6	—	—
	*O*_*43*_	—	—	—	—	6	6	5	5	—	—
*J*_*5*_	*O*_*51*_	6	7	7	6	—	—	—	—	—	—
	*O*_*52*_	—	—	—	—	—	—	—	—	8	7
	*O*_*53*_	—	—	—	—	8	7	7.7	8	—	—
*J*_*6*_	*O*_*61*_	6.8	6	7	7	—	—	—	—	—	—
	*O*_*62*_	—	—	—	—	—	—	—	—	6	6
	*O*_*63*_	—	—	—	—	8	8.4	7.6	8	—	—
*J*_*7*_	*O*_*71*_	7	5	6	7	—	—	—	—	—	—
	*O*_*72*_	—	—	—	—	—	—	—	—	6	6
	*O*_*73*_	—	—	—	—	8	7	8	8	—	—
	*O*_*74*_	—	—	—	—	6.8	6	7	7	—	—
*J*_*8*_	*O*_*81*_	5	5	4	5	—	—	—	—	—	—
	*O*_*82*_	—	—	—	—	—	—	—	—	7	7
	*O*_*83*_	—	—	—	—	8	7.7	8	7.5	—	—
	*O*_*84*_	—	—	—	—	7	7	7.5	6.5	—	—
*J*_*9*_	*O*_*91*_	8	7	8	8.2	—	—	—	—	—	—
	*O*_*92*_	—	—	—	—	—	—	—	—	6	6
	*O*_*93*_	—	—	—	—	5	6	6.5	7.2	—	—
	*O*_*94*_	—	—	—	—	5.8	7	6.6	7.2	—	—
*J*_*10*_	*O*_*101*_	7.5	9	8	6	—	—	—	—	—	—
	*O*_*102*_	—	—	—	—	7.2	8.5	8	8	—	—
	*O*_*103*_	—	—	—	—	8	8	8	8	—	—
*J*_*11*_	*O*_*111*_	6.5	7.2	6.5	8	—	—	—	—	—	—
	*O*_*112*_	—	—	—	—	7.6	9	8	7.5	—	—
	*O*_*113*_	—	—	—	—	7.8	8	7.5	8	—	—

“—” means that the operation cannot be processed on that machine.

**FIGURE 10:**
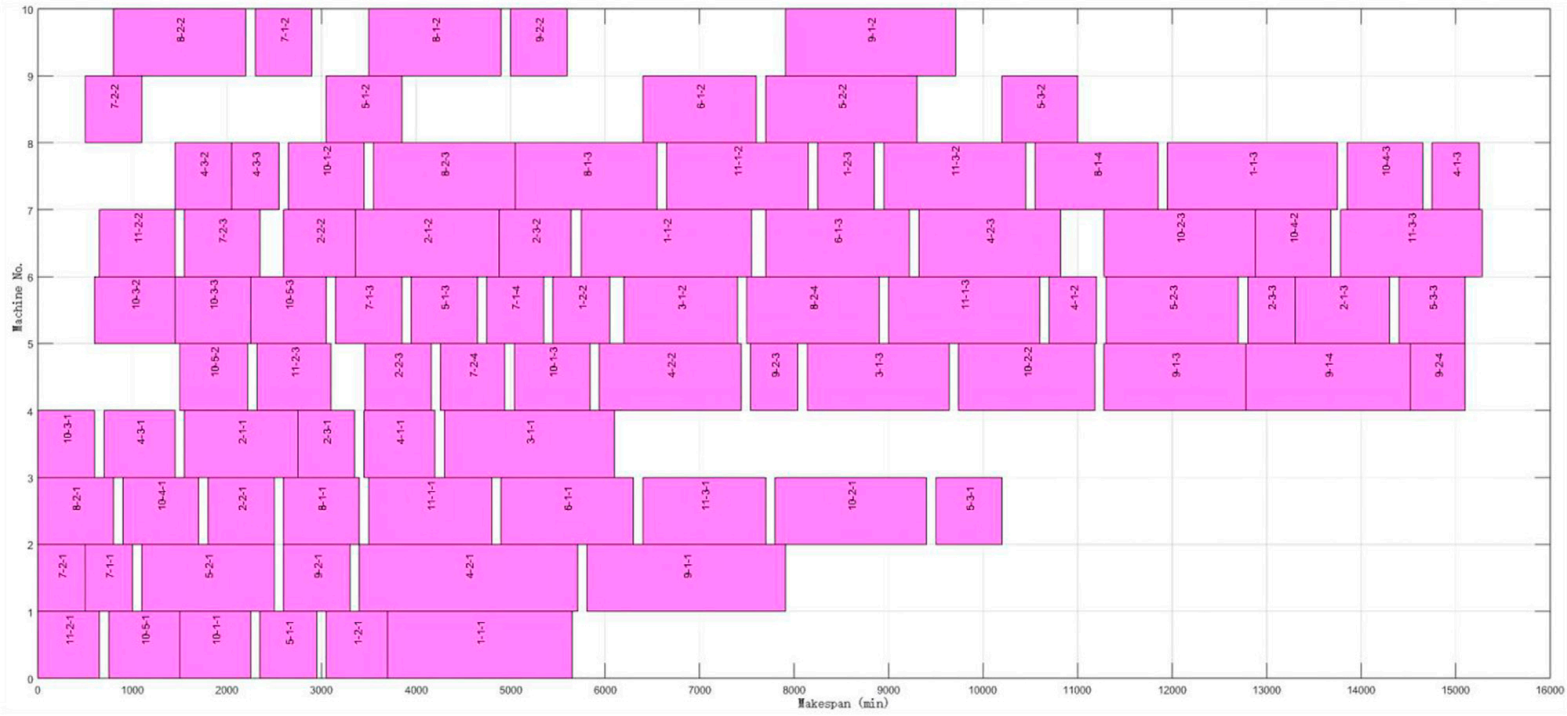
Gantt chart of lot-splitting scheduling with assembly constraints.

**FIGURE 11:**
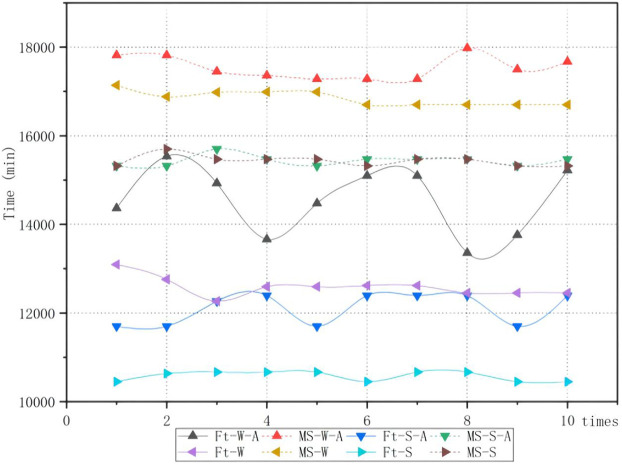
Comparison of different scenarios.

The proposed algorithm was applied to solve the problem in four scenarios classified by whether to split the job and append the assembly stage. The results are shown in Table 5. It can be found that the average flow time and makespan increase significantly when the assembly is appended. However, compared with the full batch production, batch splitting can effectively reduce the makespan and average flow time, and average efficiency can be improved by 12.14%. At the same time, the optimal makespan can be acquired even considering the assembly constraints. The Gantt chart of the optimal solution is shown in Figure 10. A comparison of experimental results is shown in Figure 11. Ft indicates that the optimization objective is an average flow time. MS indicates that the optimization objective is makespan. A stands for the assembly constraint. W and S represent scheduling with whole or splitting batches, respectively. At last, the Ft-S-A is the problem with the assembly stage and batch splitting.

**TABLE 5:** 4 × 6 lot-splitting scheduling results comparison.

Scenarios	Without assembly constraints			With assembly constraints		
	Average maximum completion time	Average flow time	Optimal maximum completion time	Average maximum completion time	Average flow time	Optimal maximum completion time
Full batch	16848	12590.1	16700	17544	14550.3	17280
Batch splitting (equal size)	15712	11823.6	15604	16361	13754.3	16147
Batch splitting (unequal size)	15433	10578.3	15320	15433	12102.7	15320
Improvement (with full batch)	8.40%	15.98%	8.26%	12.03%	16.82%	11.34%

### 4.4 Discussion

In our study, an improved algorithm based on an ABC was proposed to solve the FAJSP-LS. To the best of our knowledge, it is the first time to use the multipopulation collaboration mechanism of ABC to solve this two-stage integrated optimization problem. In examining the performance, first, the benchmark case with the minimization of makespan as the objective is used to show the optimization power on classic FJSP. Second, another modified benchmark case is used to show the algorithm’s equal and unequal splitting performance respectively. It shows unequal-size batch splitting works better than equal-size batch splitting under the flexibility situation. In addition, the proposed algorithm shows better performance on makespan compared with algorithms mentioned in the literature, because the design of a multipopulation collaboration mechanism that scouts the bee colony is in charge of batch splitting that has lower search space, and employed bee colony is in charge of batch scheduling that has a greater search space, and onlooker bee is in charge of maintaining the better solutions combined with these two subproblems. Third, the algorithm is applied to the real case in a refrigerator shell parts production workshop that produces jobs in a full batch. The experiment shows a positive impact on average flowtime and makespan by adopting LS. Compared with the original strategy, makespan can be improved by 12.03%, and average flowtime can be improved by 16.82%, significantly reducing WIP in the production and increasing the production efficiency. For this real case, unequal-size splitting also shows better performance than equal-size splitting when considering flexibility and the assembly stage.

## 5 Conclusion

This study examined an extension problem of FJSP and presented a flexible assembly job-shop scheduling problem with unequal and consistent size batch splitting, in brief, FAJSP-LS. This model comprises two subproblems: batch splitting and batch scheduling. An improved algorithm considering the multipopulation collaboration mechanism of an ABC was proposed to solve it, and it shows good performance on the problems mentioned above. Overall, it is recommended to adopt unequal-size splitting in FAJSP to optimize time-correlated objectives such as average flowtime and makespan. Furthermore, the inherent properties of multipopulation in ABC allow algorithm to solve two-stage problem without adopting distributions rules or other algorithms. A limitation of the research is that it focuses on the upper stage of the whole structure in Figure 1, and only considers the time-correlated objectives. Once the downstream assembly line stage is considered, more factors such as level scheduling that consider average demand for each component, due date on the specific workstation, transfer cost, and energy-consuming cost should be studied in the subsequent research. Furthermore, more bioinspired algorithms will be studied to optimize these kinds of multistage problems, and more neighborhood searching methods will be adopted to further the current algorithm.

## Data Availability

The original contributions presented in the study are included in the article/supplementary material. Further inquiries can be directed to the corresponding author.
